# Management of self-harm, suicidal ideation and suicide attempts

**DOI:** 10.4102/safp.v64i1.5496

**Published:** 2022-04-26

**Authors:** Adeyinka A. Alabi

**Affiliations:** 1 Department of Family Medicine, Faculty of Health Science, Walter Sisulu University, Uitenhage, South Africa; 2 Department of Family Medicine, Dore Nginza Hospital, Uitenhage, South Africa

**Keywords:** mood disorder, parasuicide, primary healthcare, psychiatric diagnosis, suicide

## Abstract

The strategic location of primary care providers (PCPs) in clinics, private general practices and emergency departments is critical to the detection and appropriate management of patients with suicidal behaviour. Their position within the primary care setting and responsibility for preventive and promotive care require PCPs to possess good clinical skills and evidence-based knowledge to assist patients presenting with suicidal ideation and behaviour. The objective of this article is to provide guidelines for the management of suicidal behaviour within the primary care setting, with the goal of reducing deaths from suicide, and the frequency and intensity of suicide attempts. The priority in the management of patients presenting at health facilities following suicide attempts is medical resuscitation and stabilisation. As soon as the patient is medically stable, a thorough suicide risk assessment, which evaluates suicidal ideation/intent, preceding circumstances, predisposing and protective factors, should be conducted. An assessment of current and ongoing suicide risk will assist in determining the safest place to manage the patient. For those with a low level of suicide risk, outpatient management may be considered in the presence of a good social support system at home and a well-documented safety plan. Measures should be put in place to address the modifiable psychosocial risk factors for suicide, whilst appropriate pharmacotherapy is instituted for co-existing mental illness. Post-discharge care such as referral to psychologist, psychiatrist or social worker should be initiated by the primary care practitioner to ensure continuity of care. Support and psycho-education should also be extended to immediate family members of patients with suicidal behaviour for their own well-being and their ability to support the patient.

## Background

Suicide is an important public health concern globally, with one person dying by suicide every 40 s, and many more making attempts to kill themselves.^1^ In South Africa, 23 people kill themselves every day, and another 460 people make attempts to end their own lives.^2^

Around 77% of people who die through suicide have regular contact with primary care providers (PCPs) in their final year before killing themselves.^3^ This window of contact offers an opportunity for PCPs to recognise and manage suicidal behaviour amongst high-risk patients, many of whom will not disclose their suicidal intent except when asked directly by a PCP.^4^

Any intervention capable of reducing the likelihood of suicide will improve the quality of care offered to at-risk patients through early detection, comprehensive assessment and effective management at the primary health care (PHC) level. The integration of all aspects of mental healthcare into primary care allows for a greater portion of the suicide-vulnerable population to be seen and assisted by PCPs.^5^ Such a goal requires that PCPs possess good knowledge of mental health and be competent in the management of common mental illnesses.^6^ Furthermore, the high prevalence of mental illness and suicidal behaviour associated with the COVID-19 pandemic^7^ requires that routine screening, assessment and management of at-risk patients become a standard of care at all health facilities.

Despite the high prevalence of successful and non-fatal suicide attempts, the requisite knowledge and confidence to prevent suicides and to render effective intervention in managing patients with suicidal behaviour are lacking amongst most PCPs.^8^ This article aims to address this gap by providing resources on the effective management of suicidal patients to reduce the intensity and occurrence of suicide and suicidal behaviour.

### Epidemiology of suicide in South Africa

In South Africa, suicide is the second leading cause of death amongst adolescents and young people between the ages of 15 years and 29 years, accounting for one in 10 unnatural deaths in this age group.^9^ The methods commonly employed are hanging, firearm and poisoning.^9^ In 2019, South Africa was ranked amongst the 10 countries with the highest number of suicides, having a total of 13 774 suicide deaths, with a male-to-female ratio of approximately 4:1.^10^

### Definition of terminology

Suicidal behaviour refers to a spectrum of behaviour that encompasses suicide ideation, non-fatal suicide attempts and suicide.^11^ Suicidal behaviour in this article refers to self-directed injurious behaviour with the intent of ending one’s life; the outcome may be fatal or non-fatal.^11^ A fatal outcome from self-directed injurious behaviour is referred to as suicide, whilst self-directed potentially injurious behaviour without fatal consequences is considered attempted suicide.^11^ The term ‘parasuicide’, a self-directed injurious behaviour without intent of death, has been replaced with ‘self-harm’ in many countries,^12^ and for the purpose of consistency, ‘self-harm’ is used in this article.

In this article, a ‘primary care practitioner’ refers to both general practitioners (not registered as a specialist with the Health Professional Council of South Africa)^13^ and healthcare professionals involved in mental healthcare services within primary care.

### Risk factors of suicidal behaviour

The non-modifiable risk factors for suicidal behaviour are previous history of suicide attempt, history of suicidal behaviour in the family, childhood adversity, previous traumas, chronic physical illnesses and gender dysphoria.^14^

A previous suicide attempt is the most important risk factor for recurrent suicide attempts and suicide.^14^ The majority of suicide attempts do not result in death, with the ratio of suicide attempts to actual suicides being 20:1^1^; however, the high prevalence of suicide attempts constitutes a great burden to emergency and mental health services.^14^

Modifiable risk factors are mental health diagnosis, medical conditions, substance abuse, financial problems, a sense of hopelessness, poor coping skills, poor problem-solving skills, previous aggressive behaviour, interpersonal relationship problems and absence of a sense of belonging.^15^ A mental health diagnosis is present in the majority of patients with suicidal behaviour but is often unrecognised and remains untreated by primary health providers.^16,17^

## Management of patients with suicidal behaviour

### Acute management

Patients with suicide attempts who present at the emergency department immediately post-attempt may require thorough medical evaluation, resuscitation and stabilisation of their medical condition. Primary care providers should be aware of the common methods of suicide attempts in their area of practice to ensure prompt and appropriate intervention in such cases. Self-poisoning is the commonest method of suicide attempt in South Africa, with the most commonly ingested substances being paracetamol, household chemicals and antiretroviral drugs.^18^ The protocol for the treatment of patients who have consumed these substances should be easily accessible to all staff of emergency units. Furthermore, practitioners should be encouraged to consult a poison information centre if uncertain on how to manage the patient. Once the patient is medically stable, a suicide risk assessment should be conducted, along with a detailed psychiatric evaluation. In addition, a clear management plan for the prevention of future suicide attempts should be worked out with the patient and immediate family.

### Evaluation for suicidal ideation and self-harm amongst the at-risk group

Primary care providers should assess the level of suicide risk for every patient who has presented with a suicide attempt, by conducting a thorough assessment of the patient’s mental and medical health status, family history and psychological, social and environmental problems.^19^ The overall risk category (low, intermediate or high) will determine the safest environment to best manage the patient during the crisis. However, consideration should be given to the least restrictive environment. A patient categorised as low risk, with good home support and without active suicidal thoughts and planning, should be managed on an outpatient basis.^20^ This should be properly negotiated and discussed with the patient and family members. However, if the provider is in doubt of the level of risk, or there is poor support at home, the patient should be considered for in-hospital management. The healthcare provider’s approach to the patient during this encounter should be non-judgemental, reassuring and comforting, characterised by empathic listening and the stimulation of hope.^21^

Follow-up out-patient management should be clearly communicated to the patient and should include referral for both social support and psychological therapy to improve the patient’s problem-solving and coping skills. Primary care providers should explain the benefits and effectiveness of various modalities of psychotherapy in reducing suicidal behaviour to the patient and their family members. Short interval scheduled clinic appointments should be put in place following the initiation of psychotherapy or pharmacotherapy for close monitoring, because of a temporarily increased risk of suicide that may be associated with treatment.^22^ Furthermore, a system should be put in place to track those patients who miss their follow-up appointments.

The high prevalence of mental illness, especially mood disorders, substance use disorders (SUD), eating disorders, psychosis and cluster-B traits amongst patients with suicide deaths necessitates that PCPs painstakingly screen for symptoms of mental illness in anyone displaying suicidal behaviour.^23^ Depressive disorders, which account for more than 50% of mental illnesses amongst patients with suicidal behaviour, should be promptly diagnosed and treated. Patients with moderate-to-severe depression or those with poor improvement to psychotherapy should be considered for pharmacological treatment.^24^ When initiating antidepressants, consideration should be given to the safety profile of medication, alongside other patient-related factors. Primary care providers should prescribe selective serotonin reuptake inhibitors (SSRI) because of their improved safety profile over tricyclic antidepressants, particularly in overdose. Furthermore, the supply of medication to suicidal patients should be limited to a few days or a few weeks at a time to restrict the amount of drugs available for overdose.^25^ Patients who show poor response to medications or who persist with suicidal behaviour despite treatment should be referred to a psychiatrist for further management.

Primary care providers should also routinely screen for SUD amongst patients with suicidal behaviour and offer them brief counselling, referral for rehabilitation and pharmacotherapy – depending on the severity of the SUD.^26^

Particular attention should be paid to suicidal behaviour in patients with bipolar disorder because of a higher rate of completed suicide amongst this population as compared to patients with other psychiatric diagnoses.^27^ Lithium has anti-suicidal properties and should be considered in the management of bipolar patients at high risk of suicide.^28^ Similarly, clozapine pharmacotherapy reduces suicidal behaviour amongst patients with schizophrenia and schizoaffective disorders.^29^ However, there is a set of other criteria which patients must meet before consideration is given to either lithium or clozapine, and a psychiatric consultation should be sought by PCPs in such cases.

Primary care providers should provide psycho-education to all of their patients on coping skills, available mental health resources, social support structures available and whom to contact whenever they are in a psychological crisis. In conjunction with the patient, a PCP should develop a well-documented, stepwise safety plan for every suicidal patient that includes recognising warning signs; adopting self-initiated coping strategies; turning to family and friends for support; limiting access to drugs and alcohol; restricting access to lethal means; and knowing the contact details of PCPs, emergency departments and crisis agencies.^30^

### High risk of suicide category

A patient rated as having a moderate to high level of suicide risk from comprehensive suicide risk assessment, or who has severe depression, a heightened sense of hopelessness, severe SUD or psychosis should be referred to a mental health facility via the emergency unit of the hospital for admission. Suicide risk assessment tools, such as Beck’s Depression Inventory (BDI), Beck’s Hopelessness Scale (BHS), the SADPERSONS scale and others, are available for use in various clinical settings to inform suicide risk assessment. However, these tools are unreliable and inaccurate in distinguishing high-risk and low-risk patients^31^ and must be used in conjunction with clinical gestalt.

A patient with severe suicidal behaviour should always be in the company of a family member or a health worker and should never be left alone at any time (i.e. suicide watch).

If such a patient declines admission, an involuntary admission under the *Mental Health Act* of 2002 should be instituted to prevent the patient from committing suicide. Proper handover from one end of the chain to another (i.e. from the PCP to emergency medical service personnel, and then to the staff of the emergency department) is important for the effective management of suicidal patients.

In addition, providers must keep adequate documentation of every encounter with the patient, paying special attention to the presenting complaints, sequence of events, risk assessment, management and follow-up plan.

In conclusion, PCPs have a central role to play in the early diagnosis and management of patients with suicidal behaviour. The use of a comprehensive approach to patients’ consultations will promote a therapeutic alliance with the patient and allow PCPs to address the many factors that make patients vulnerable to suicidal behaviour. The PCP’s biopsychosocial management of the patient should include the psycho-education of the patient and families, psychotherapy to help with coping and problem-solving skills, social support and pharmacotherapy.
